# West Nile Virus Challenge Alters the Transcription Profiles of Innate Immune Genes in Rabbit Peripheral Blood Mononuclear Cells

**DOI:** 10.3389/fvets.2015.00076

**Published:** 2015-12-14

**Authors:** Muhammad J. Uddin, Willy W. Suen, Natalie A. Prow, Roy A. Hall, Helle Bielefeldt-Ohmann

**Affiliations:** ^1^School of Veterinary Science, University of Queensland, Gatton, QLD, Australia; ^2^QIMR Berghofer Medical Research Institute, Brisbane, QLD, Australia; ^3^Australian Infectious Diseases Research Centre, University of Queensland, St Lucia, QLD, Australia; ^4^School of Chemistry and Molecular Biosciences, University of Queensland, St Lucia, QLD, Australia

**Keywords:** West Nile virus, rabbit, PBMCs, innate immune response, cytokines, TLRs

## Abstract

The peripheral innate immune response to West Nile virus (WNV) is crucial for control of virus spread to the central nervous system. Therefore, transcriptomes encoding the innate immune response proteins against WNV were investigated in peripheral blood mononuclear cells (PBMCs) of New Zealand White rabbits, a recently established novel rabbit model for WNV pathogenesis studies. PBMCs were challenged with an Australian WNV strain, WNV_NSW2011_, *in vitro*, and mRNA expression of selected immune response genes were quantified at 2-, 6-, 12-, and 24-h post-infection (pi) using qRT-PCR. Compared to mock-inoculated PBMCs, WNV-stimulated PBMCs expressed high levels of interferon (IFN) alpha (IFNA), gamma (IFNG), IL6, IL12, IL22, CXCL10, and pentraxin 3 (PTX3) mRNA. Likewise, TLR1, 2, 3, 4, 6, and 10 mRNA became up-regulated with the highest expression seen for TLR3, 4, and 6. TLRs-signaling downstream genes (MyD88, STAT1, TRAF3, IRF7, and IRF9) subsequently became up-regulated. The high expression of IFNs, TLR3, TLR4, TRAF3, STAT1, IRF7, and IRF9 are in accordance with antiviral activities, while expression of TNFA, HO1, iNOS, caspase 3, and caspase 9 transcripts suggests the involvement of oxidative stress and apoptosis in WNV-stimulated rabbit PBMCs, respectively. The level of WNV_NSW2011_ RNA increased at 24-h pi in PBMCs challenged with virus *in vitro* compared to input virus. The expression dynamics of selected genes were validated in PBMCs from rabbits experimentally infected with WNV *in vivo*. Higher expression of IFNA, IFN beta (IFNB), IFNG, TNFA, IL6, IL22, PTX3, TLR3 and TLR4, IRF7, IRF9, STST1, TRAF3, caspase 3, and caspase 9 were seen in PBMCs from WNV-infected rabbits on day 3 post-intradermal virus inoculation compared to PBMCs from uninfected control rabbits. This study highlights the array of cytokines and TLRs involved in the host innate immune response to WNV in the rabbit leukocytes and suggests that these cells may be a useful *in vitro* model for WNV infection study.

## Introduction

Since the first isolation of the Australian strain of West Nile virus, the Kunjin strain (WNV_KUN_), in 1960 in North Queensland (1), it has been found to be endemic in Australia (2). WNV_KUN_ belongs to lineage 1 of WNV, which also includes the highly pathogenic neuro-invasive New York 99 strain (WNV_NY99_) (3). WNV_KUN_ causes mainly asymptomatic infection and only a small number of mild human cases have been documented with no reported fatalities in Australia (2). In contrast, since its introduction into New York in 1999, WNV_NY99_ has spread rapidly throughout the USA with close to 40,000 human cases of WNV disease and more than 1,600 deaths reported in the USA between 1999 and 2014 (4). WNV has also spread to other parts of the Americas, and also to Europe, Asia, and the Middle East (5). In addition to human infections, the WNV_NY99_ virus has caused significant morbidity and mortality in horses and birds, with more than 20,000 equine cases and hundreds of thousands of avian deaths (6). Relative to the WNV_NY99_ strain, WNV_KUN_ exhibits much reduced virulence in humans, animals, and birds (2). Equine disease associated with WNV_KUN_ infection was rare, however, in early 2011 following extensive flooding in the Murray–Darling river basin and other inland river systems, an unprecedented outbreak of equine encephalitis occurred in south-eastern Australia involving more than 1,000 horses (7). Genomic sequencing of viruses isolated from a horse succumbing to encephalitis in the 2011 outbreak revealed the etiological agent to be a variant strain of WNV, most closely related to WNV_KUN_ and subsequently named WNV_NSW2011_ (7). As the virus strain may still be circulating and the human and equine populations in Eastern Australia remain susceptible to WNV_KUN_ (8, 9), it is important to get a better understanding of the host response to this virus.

Peripheral blood mononuclear cells (PBMCs) are among the first immune components to encounter WNV following a mosquito bite. It has been postulated that the PBMC may serve as target cells for initial replication of WNV and play a role in subsequent viral dissemination (10). Additionally, primary PBMC cell culture has been proposed to be a potentially useful model of a natural WNV host (10). To survive virus infection, the host must recognize invasion and develop an effective antiviral immune response. This response is initiated in infected cells after detection of non-self pathogen-associated molecular patterns (PAMPs). PAMPs motifs are detected by specific, conserved host molecular patterns – pathogen recognition receptors (PRRs), such as Toll-like receptors (TLRs), which trigger signaling cascades that induce the activation of interferon (IFN) regulatory factors (IRFs) and nuclear factor kappa B (NFκB), leading to expression of antiviral molecules, including type I IFNs (IFNA and IFNB), and hundreds of different IFN-stimulated effector genes (ISGs). ISG products include additional antiviral effector molecules and immunomodulatory cytokines that serve to restrict virus replication and modulate the immune response (11).

The signaling pathways thought to detect entry and infection by WNV and initiate a protective IFN response have mostly been studied in mice (12–18) with only limited studies in the horse (19). Based on those studies, a partial signaling pathway has been proposed by which WNV and other flaviviruses are detected, and the effector mechanisms that contribute to protective cell-intrinsic immunity executed (11, 20). Nevertheless, there appears to be some discrepancies between the protective immune mechanisms between humans and mice (21, 22) and even more so between mice and horses (23, 24). In order to overcome the latter problem, we have recently established a New Zealand White (NZW) rabbit model for WNV-infection (25). Physiologically, rabbits resemble horses by being hindgut fermenters and 10–20% of feral rabbits may be exposed to WNV in any season throughout Australia (8). Previously, gene expression has been performed in selected tissues (thalamus and cerebrum) of horse (19), mouse (26), NZW rabbits (25), and in human cells and tissues infected with WNV (27). However, there is a gap in knowledge of the expression patterns of immune-related genes during the early time points following infection. Therefore, this study aimed to characterize the innate immune response in rabbit PBMCs, exposed *in vitro* to WNV_NSW2011_ using transcriptomic analysis of immunologically important genes. Selected transcripts were subsequently confirmed in the *in vivo* WNV-infection model, supporting the contention that *in vitro* studies of PBMCs responses are a useful surrogate model for acute WNV infection in natural hosts and relevant animal model.

## Materials and Methods

### Preparation of WNV

The WNV_NSW2011_ strain used in this study was isolated from a 10% weight/volume brain homogenate of an encephalitic horse during the 2011 Australian arboviral outbreak. The virus isolate was passaged initially in C6/36 cells (*Aedes albopictus* mosquito cells), followed by 1× in Vero (African green monkey kidney) cells and 3× final passages in C6/36 cells cultured with 10% fetal bovine serum (FBS) at 28°C before use. Detailed characterization of the mouse virulence of WNV_NSW2011_ has been described in Frost et al. (7). The mock inoculum consisted of tissue culture medium only. The virus stock was stored at −80°C. The titer of the stock was quantified using standard plaque assay on Vero cells, as described previously (25, 28). The WNV_NSW2011_ was diluted to 1 × 10^6^ PFU/50 μL for use in PBMCs infection *in vitro* experiments.

### PBMCs Isolation, Culture, and Challenged with Viruses

EDTA-stabilized blood samples were collected from WNV-seronegative NZW rabbits (*n* = 3) (25). All animal procedures had received prior approval from the University of Queensland Animal Ethics Committee (SVS/369/12/ARC), and all procedures were performed under xylazine sedation and anesthesia (25). Blood was collected by cardiac bleed of anesthetized rabbits into EDTA-coated collection tubes (Vacuette^®^, Greiner Bio One, Australia) immediately prior to their euthanasia. The PBMCs were isolated from the blood using Ficoll-Histopaque (Sigma) as described previously (29). The viability of the purified PBMCs was assessed using the trypan blue exclusion method and was always >90%. The cells were counted using a hemocytometer and the concentration was adjusted to 1 × 10^6^ cells/0.5 ml in RPMI-1460 supplemented with l-glutamine (2 nM), streptomycin (50 μg/mL), penicillin (50 U/mL), and 10% FBS (29). The cells were cultured in 24-well cell culture plates (Costar Corning, the Netherlands) seeded with 500 μL of cell suspension per well and incubated at 37°C with 5% CO_2_. Remaining PBMCs (fresh-isolated PBMCs) were pelleted and kept at −80°C for RNA isolation. After 1 h of incubation, the PBMCs were challenged by adding 50 μL of WNV_NSW2011_ to each well to give a final multiplicity of infection (MOI) of one. The dose of virus was chosen based on the typical dose inoculated by mosquitoes (30). Additional 450 μL medium was added to each well to make up the final volume of 1 ml. One well of PBMCs per animal was harvested at 2, 6, 12, and 24 h after virus infection, respectively. The harvested cells (WNV-stimulated PBMCs) were pelleted and kept at −80°C until subjected to RNA isolation. For complete harvesting of adherent PBMCs, detachment with lidocaine HCl (12 mM) was performed (31). To ensure the complete harvesting of cells, wells were checked using an inverted microscope. Duplicate wells of uninfected cells (mock-inoculated PBMCs) were cultured in 500 μL of RPMI culture media supplemented with 10% FBS. They were harvested and treated in a similar manner at each time point.

### PBMCs Isolation from Infected Rabbits

In order to compare the transcriptional profile of in vitro challenged PBMCs against those from in vivo challenged rabbits blood samples were obtained from WNV_NSW2011_-infected New Zealand White (NZW) rabbits (*n* = 3) on day 3 pi, and mock-infected NZW rabbits (*n* = 3) on day 6 post-mock (medium only) inoculation (25). Collected blood was immediately processed for PBMCs isolation, as described above.

### RNA Isolation and Transcriptome Quantification

Total RNA was isolated from PBMCs using miRNeasy RNA isolation kit (Qiagen Pty Ltd., Australia) and on-column DNA digestion (Qiagen) was performed following the manufacturer’s instructions. The quantity and quality of RNA was measured using NanoDrop 1000 spectrophotometer (Thermo Scientific, Australia). The isolated RNA was subjected to PCR with GAPDH primers without a reverse transcription step and run in gel to check for DNA-contamination. None was found to be contaminated (data not shown). The isolated RNA was kept at −80°C for further transcriptome analysis using quantitative real time PCR (qRT-PCR). Primers for cytokines, TLRs and downstream genes, apoptosis and oxidative stress-related genes, and two normalizer genes (*GAPDH* and *PPIA*) were designed from FASTA products of the GenBank mRNA sequences for *Oryctolagus cuniculus* using the Primer3 program (32). No suitable normalizer genes has been reported in rabbits PBMCs yet, but *GAPDH* and *PPIA* are reported to be appropriate stably expressed normalizer genes in PBMCs in pigs (33). The WNV_NSW2011_ (WNV_KUN)_-specific primers (34) and primers of some cytokines (35) were described earlier. Details of the primers are given in Table 1.

**Table 1:** **List of primer sequences used in this study**.

Gene	Primer set^a^	Amplicon size (bp)	GenBank accession number
*Kunjin*	F: AACCCCAGTGGAGAAGTGGA^b^	70	D00246
	R: TCAGGCTGCCACACCAAA		
*IFNA*	F: TGCTTGCAGGACAGACATGA	95	XM_002708065.1
	R: ATCTCGTGGAGCACAGAGAT		
*IFNB*	F:TCCAACTATGGCACGGAAGTCT^c^	89	XM_002707968
	R:TTCTGGAGCTGTTGTGGTTCCT		
*IFNG*	F:TGCCAGGACACACTAACCAGAG^c^	127	NM_001081991
	R:TGTCACTCTCCTCTTTCCAATTCC		
*TNFA*	F:CTGCACTTCAGGGTGATCG^c^	94	NM_001082263
	R:CTACGTGGGCTAGAGGCTTG		
*IL6*	F: CTACCGCTTTCCCCACTTCAG^c^	135	NM_001082064.2
	R: TCCTCAGCTCCTTGATGGTCTC		
*IL12/IL23P40*	PS211: CTCCGAAGAAGATGGCATTACC^c^	126	XM_002710347
	PS212: TCTCCTTTGTGGCAGGTGTATTG		
*IL22*	PS567: ACCTCACCTTCATGCTGGCTAA	84	XM_002711248
	PS568: CATGGAACAGCTCATTCCCAAT		
*CXCL10*	F: ATAGAAGCATCCTGAGCCCA	86	XM_002717106.1
	R: GAACTGCAAACTGAGGCCAA		
*PTX3*	F: TTCCCCATGCGTTCCAAGAA	95	XM_002716328.1
	R: GTGGCTTTGACCCAAATGCA		
*HO1*	F: ACTGCCGAGGGTTTTAAGCT	88	XM_002711415.1
	R: GGTTCTCCTTGTTGTGCTCA		
*iNOS*	F: GACGTCCAGCGCTACAATATCC^c^	102	XM_002718780
	R: GATCTCTGTGACGGCCTGATCT		
*Caspase 3*	F: AAGCCGACTTCCTGTATGCA	111	NM_001082117.1
	R: CGTACTCTTTCAGCATGGCA		
*Caspase 9*	F: AAACGTGGATTTGGCGTACG	80	XM_002722329.1
	R: TGCTGCTGAAGTTCACGTTG		
*TLR1*	F: TGTGTCCCACAATGAGCTGT	93	XM_002709270.1
	R: GGCAGAGCATCAAACGCATT		
*TLR2*	F: GCTGCGCAAGATCATGAACA	96	NM_001082781.1
	R: TTTATGGCGGCCCTCAAGTT		
*TLR3*	F: ATGACCTGCCCACCAACATA	140	NM_001082219.1
	R: TTCTGGCTCCAGCTTTGAGA		
*TLR4*	F: AGGCTGTTGGTGGAAGTTGA	91	NM_001082732.2
	R: TGCTTATCTGACAGGTGGCA		
*TLR6*	F: CATTGAGCACAACGCAGTGT	108	XM_002709388.1
	R: AGCTCGCATGTACAGTGGAA		
*TLR10*	F: ACACCGGTAATGCACTTGGA	85	XM_002709387.1
	R: TAAGCAAGGTGTCTGGCCAT		
*MyD88*	F: GCCAGTGAGCTCATCGAGAA	80	XM_002723869.1
	R: TCACACTCCTTGCTCTGCAG		
*IRF1*	F: AGCACTGTCACCACATAGCA	120	NM_001171347.1
	R: TCATCTGTCGCAGCTTCAGA		
*IRF7*	F: AAGTGCAAGGTGTACTGGGA	119	XM_002724304.1
	R: AGCTCTTGGAAGAAGGTGCT		
*IRF9*	F: TAACTGAGGCTGCTGTGCAA	103	XM_002718097.1
	R: ACACGCCCGTTGTAGATGAA		
*TRAF3*	F: TGGCTATAAGATGTGCGCCA	95	XM_002721716.1
	R: ACTCTCCACGCATGATGACA	
*STAT1*	F: TTCAACATCCTGGGCACACA	112	XM_002712346.1
	R: TGCCAGCGTTCTTCTGTTCT		
*GAPDH*	F: TGACGACATCAAGAAGGTGGTG**	126	NM_001082253
	R: GAAGGTGGAGGAGTGGGTGTC		
*PPIA*	F: AGGGCATGAGCATTGTGGAA	86	NM_001082057.1
	R: TCCACAGTTGGCAATGGTGA		

*^a^Annealing temperature was 60°C for all the primer sets*.

*^b^Primer set is adopted from Ref. (34)*.

*^c^Primer set are adopted from Ref. (35)*.

**Figure 1:**
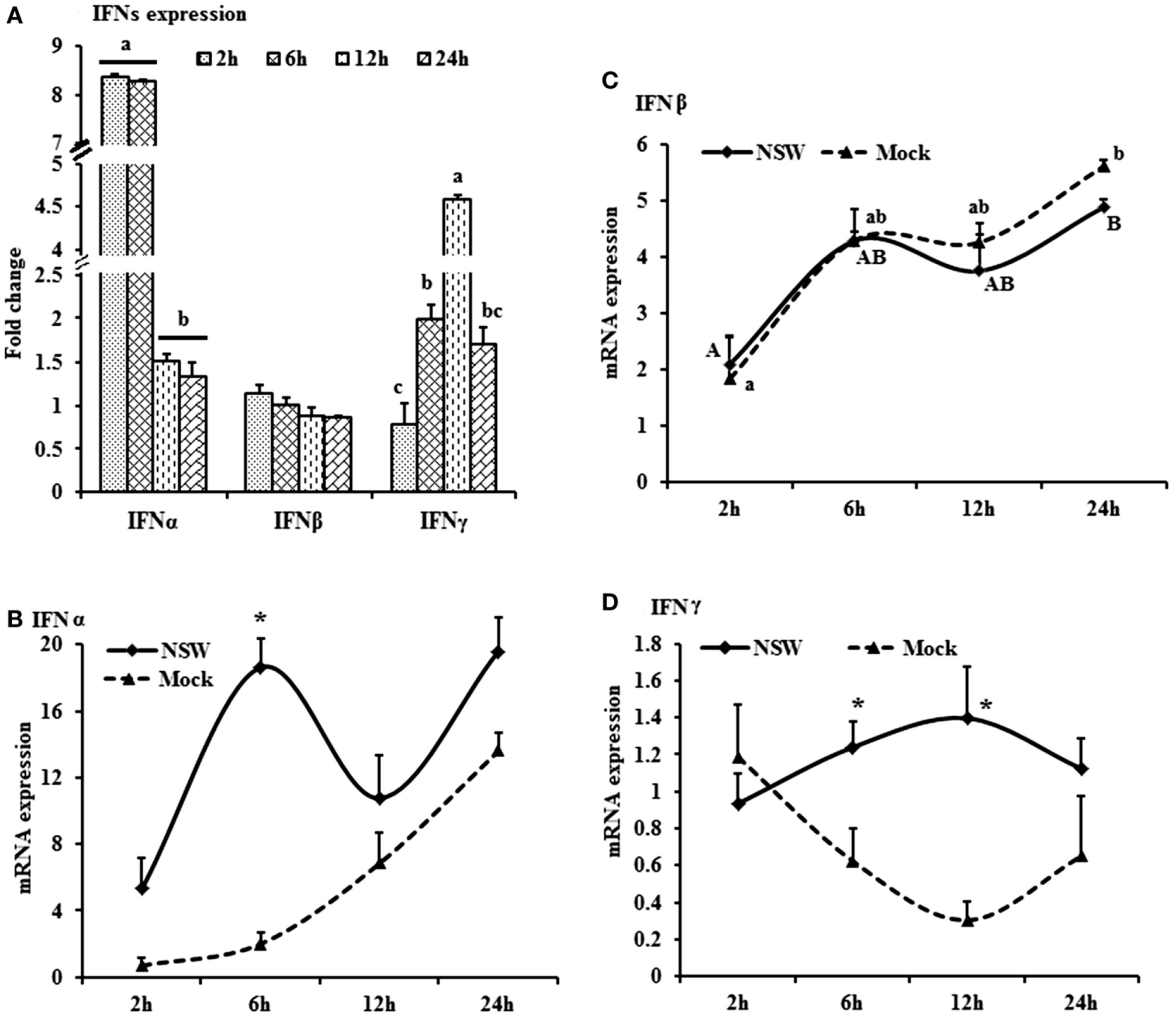
**Differential expression of interferon genes in response to West Nile virus**. **(A)** Interferons mRNA expression in fold change. The ΔΔCt (ΔΔCt = ΔCt_WNV_ − ΔCt_mock_) values were calculated by subtracting the ΔCt of genes in mock-inoculated PBMCs (*n* = 3). The bar graph showed the expression of genes in WNV-infected PBMCs over mock-inoculated PBMCs (fold change: the normalized expression value of a gene in WNV-stimulated cells/the normalized expression value of a gene in mock-inoculated cells). Bars without common superscripts (a,b; a,c; b,c) denote statistical difference among time points (*p* < 0.05). **(B–D)** Relative expression of interferons mRNA, accounting for the effects of culture conditions on gene transcription in WNV- and mock-inoculated rabbit PBMCs (*n* = 3). To compare the normalized expression of IFNs genes from PBMCs harvested at each time point to their respective expression levels before either WNV inoculation or mock inoculation, the ΔΔCt values were calculated by subtracting ΔCt of genes in fresh-isolated PBMCs from the ΔCt of genes in WNV- or mock- inoculated PBMCs at each time-point (for WNV-stimulated PBMCs, ΔΔCt_WNV_ = ΔCt_WNV_ − ΔCt_fresh_; and for mock-inoculated PBMCs, ΔΔCt_mock_ = ΔCt_mock_ − ΔCt_fresh_). A time-dependent relative expression patterns of **(B)** *IFNA*, **(C)** *IFNB*, and **(D)** *IFNG* mRNA in WNV-stimulated rabbit PBMCs at different time points. Line graphs without common superscript differ significantly (*p* < 0.05). Upper case letter denotes difference of a gene expression among the time points in WNV-challenged cells; lower case letter denotes difference of a gene expression among the time points in mock-inoculated cells. *indicates the difference of a gene expression between WNV- and mock-challenged cells in the same time point (*p* < 0.05).

**Figure 2:**
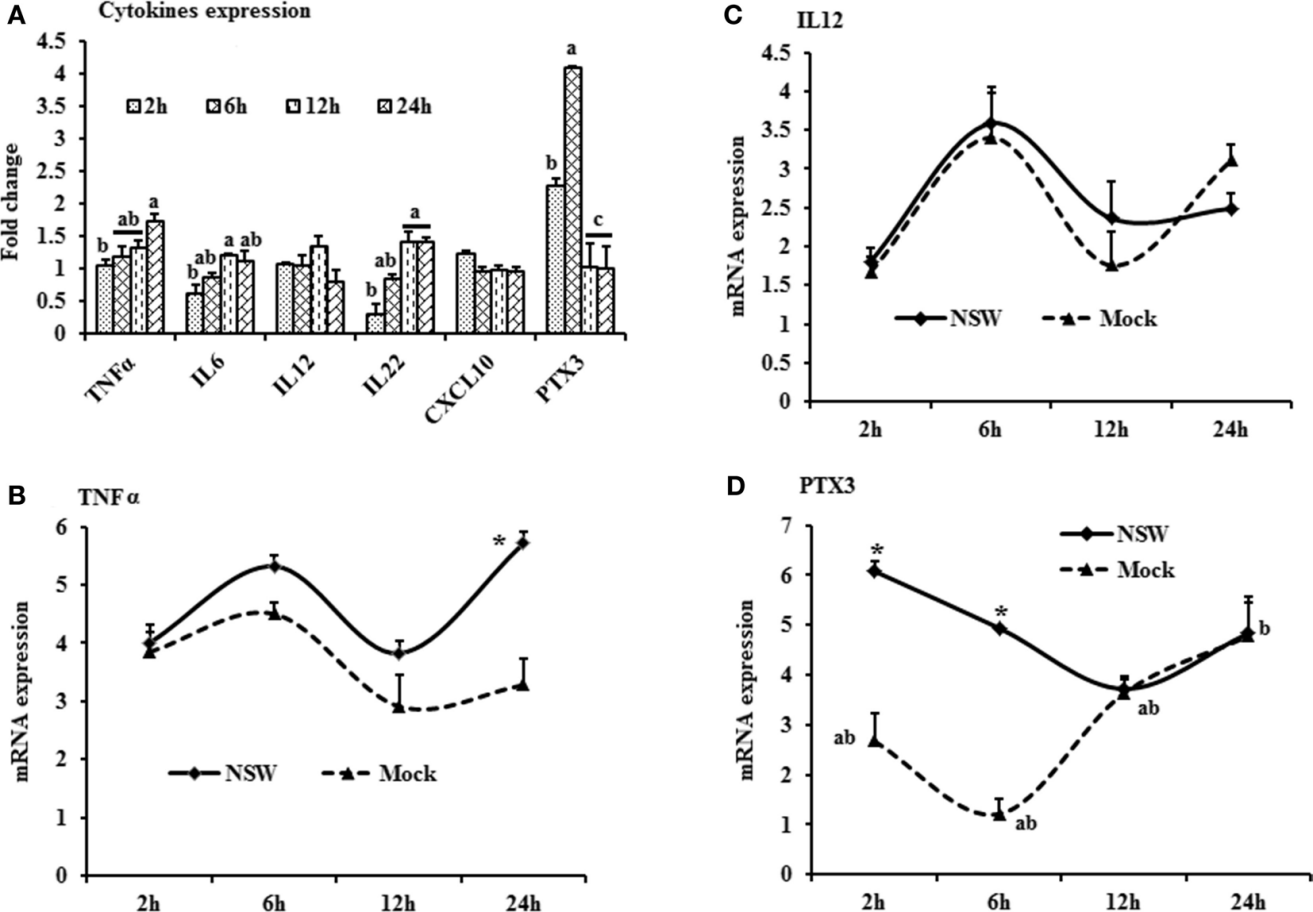
**Differential expression of inflammatory cytokine genes in response to West Nile virus**. **(A)** Cytokines mRNA expression in fold change. The ΔΔCt (ΔΔCt = ΔCt_WNV_ − ΔCt_mock_) values were calculated by subtracting the ΔCt of genes in mock-inoculated PBMCs (*n* = 3). The bar graph showed the expression of genes in WNV-infected PBMCs over mock-inoculated PBMCs (fold change: the normalized expression value of a gene in WNV-stimulated cells/the normalized expression value of a gene in mock-inoculated cells). Bars without common superscripts (a,b; a,c; b,c) denote statistical difference among time points (*p* < 0.05). **(B–D)** Relative expression of cytokines mRNA, accounting for the effects of culture conditions on gene transcription in WNV- and mock-inoculated rabbit PBMCs (*n* = 3). To compare the normalized expression of cytokine genes from PBMCs harvested at each time point to their respective expression levels before either WNV inoculation or mock inoculation, the ΔΔCt values were calculated by subtracting ΔCt of genes in fresh-isolated PBMCs from the ΔCt of genes in WNV- or mock-inoculated PBMCs at each time-point (for WNV-stimulated PBMCs, ΔΔCt_WNV_ = ΔCt_WNV_ − ΔCt_fresh_; and for mock-inoculated PBMCs, ΔΔCt_mock_ = ΔCt_mock_ − ΔCt_fresh_). A time-dependent relative expression patterns of **(B)***TNFA*, **(C)** *IL12*, and **(D)** *PTX3* mRNA in WNV-challenged rabbit PBMCs at different time points. Line graphs without common superscript differ significantly (*p* < 0.05). Upper case letter denotes difference of a gene expression among the time points in WNV-challenged cells; lower case letter denotes difference of a gene expression among the time points in mock-inoculated cells. *indicates the difference of a gene expression between WNV- and mock-challenged cells in the same time point (*p* < 0.05).

**Figure 3:**
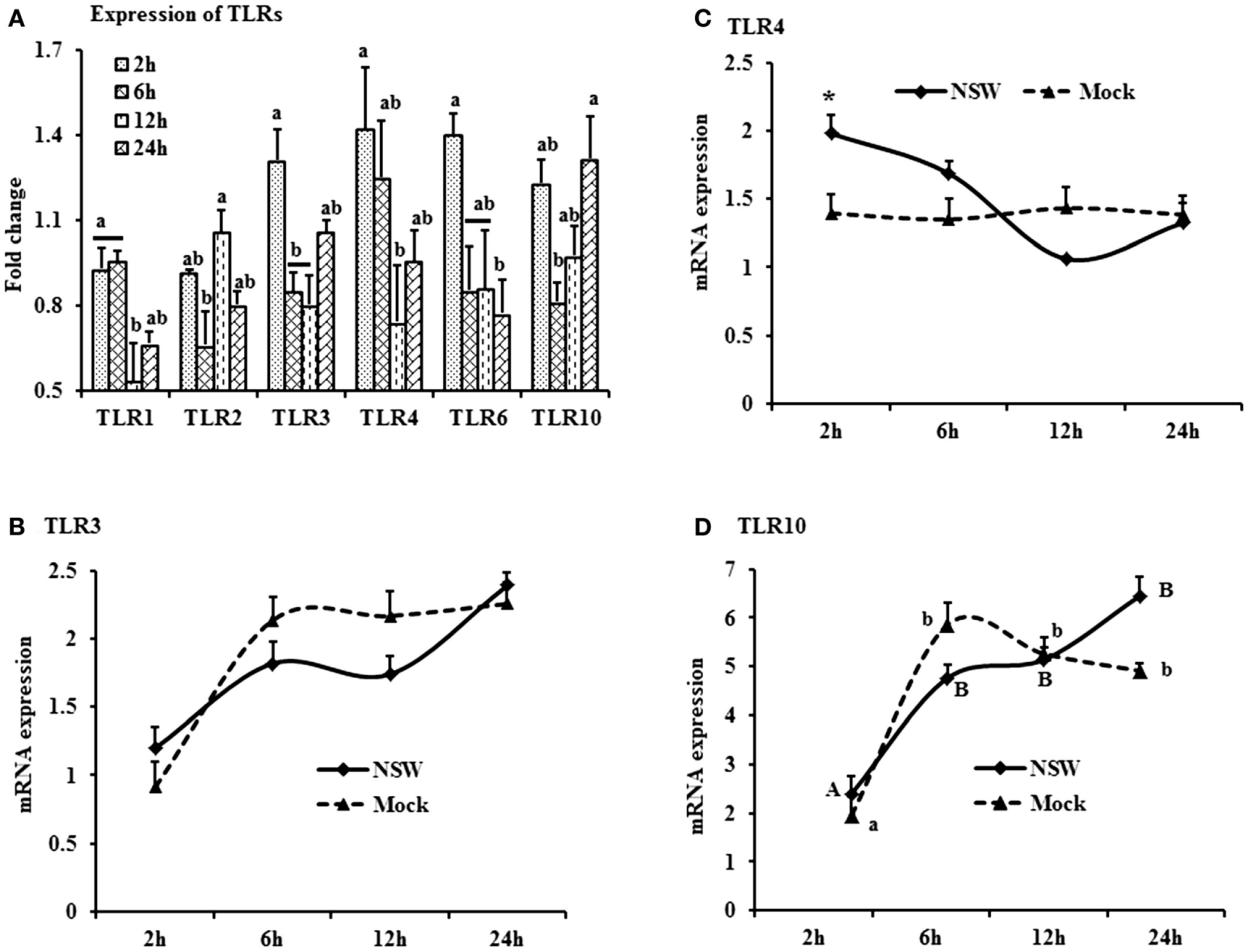
**Differential expression of Toll-like receptor genes in response to West Nile virus**. **(A)** TLrs mRNA expression in fold change. The ΔΔCt (ΔΔCt = ΔCt_WNV_ − ΔCt_mock_) values were calculated by subtracting the ΔCt of genes in mock-inoculated PBMCs (*n* = 3). The bar graph showed the expression of genes in WNV-infected PBMCs over mock-inoculated PBMCs (fold change: the normalized expression value of a gene in WNV-stimulated cells/the normalized expression value of a gene in mock-inoculated cells). Bars without common superscripts (a,b; a,c; b,c) denote statistical difference among time points (*p* < 0.05). **(B–D)** Relative expression of TLRs mRNA, accounting for the effects of culture conditions on gene transcription in WNV- and mock-inoculated rabbit PBMCs (*n* = 3). To compare the normalized expression of TLRs genes from PBMCs harvested at each time point to their respective expression levels before either WNV inoculation or mock inoculation, the ΔΔCt values were calculated by subtracting ΔCt of genes in fresh-isolated PBMCs from the ΔCt of genes in WNV- or mock-inoculated PBMCs at each time-point (for WNV-stimulated PBMCs, ΔΔCt_WNV_ = ΔCt_WNV_ − ΔCt_fresh_; and for mock-inoculated PBMCs, ΔΔCt_mock_ = ΔCt_mock_ − ΔCt_fresh_). A time-dependent relative expression patterns of **(B)** *TLR3*, **(C)** *TLR4*, and **(D)** *TLR10* mRNA in WNV-challenged rabbit PBMCs at different time points. Line graphs without common superscript differ significantly (*p* < 0.05). Upper case letter denotes difference of a gene expression among the time points in WNV-challenged cells; lower case letter denotes difference of a gene expression among the time points in mock-inoculated cells. *indicates the difference of a gene expression between WNV- and mock-challenged cells in the same time point (*p* < 0.05).

**Figure 4:**
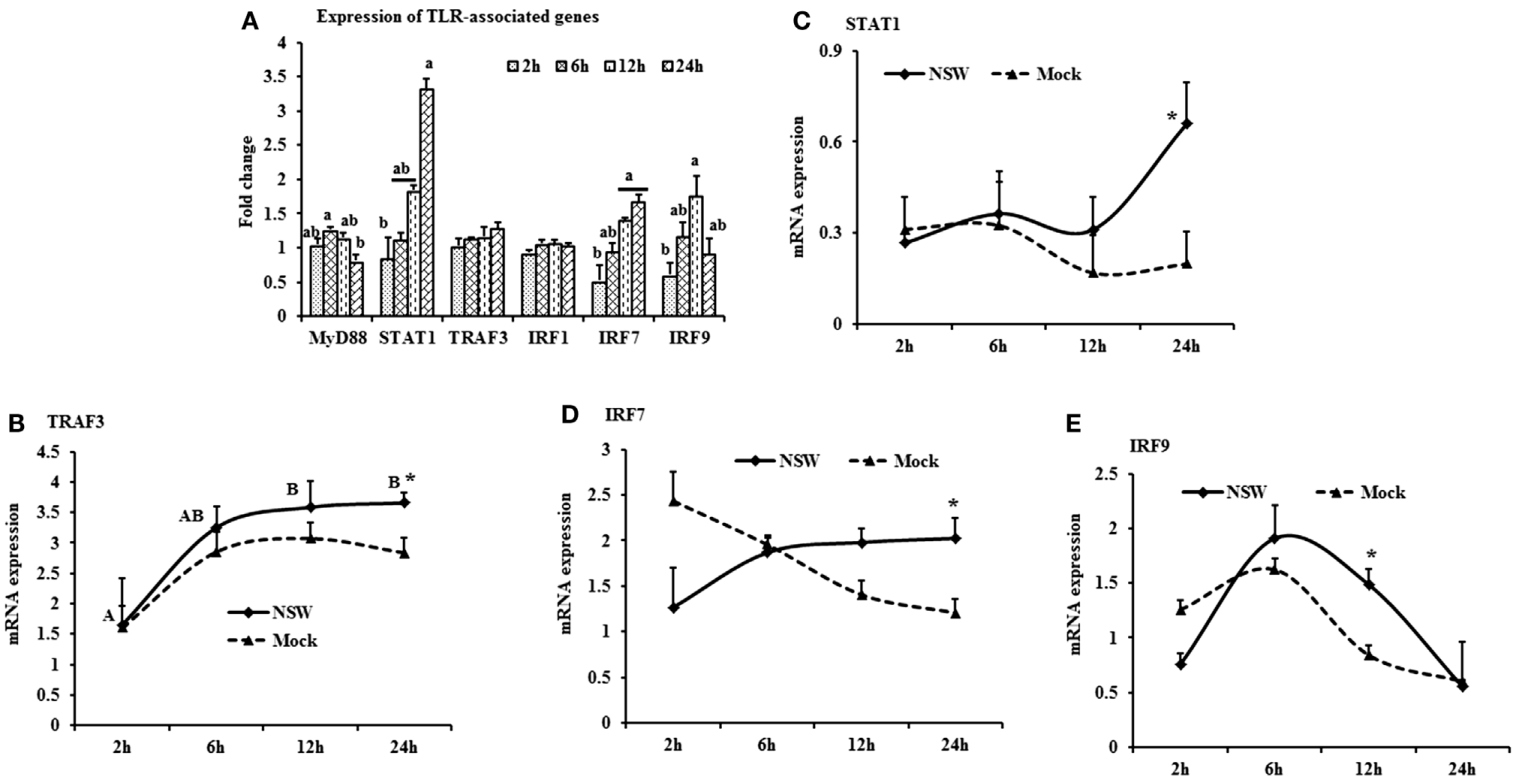
**Differential expression of the TLR downstream genes in response to West Nile virus**. **(A)** TLR-associated genes mRNA expression in fold change. The ΔΔCt (ΔΔCt = ΔCt_WNV_ − ΔCt_mock_) values were calculated by subtracting the ΔCt of genes in mock-inoculated PBMCs (*n* = 3). The bar graph showed the expression of genes in WNV-infected PBMCs over mock-inoculated PBMCs (fold change: the normalized expression value of a gene in WNV-stimulated cells/the normalized expression value of a gene in mock-inoculated cells). Bars without common superscripts (a,b) denote statistical difference among time points (*p* < 0.05). **(B–E)** Relative expression of TLRs-associated genes mRNA, accounting for the effects of culture conditions on gene transcription in WNV- and mock-inoculated rabbit PBMCs (*n* = 3). To compare the normalized expression of TLR-associated genes from PBMCs harvested at each time point to their respective expression levels before either WNV inoculation or mock inoculation, the ΔΔCt values were calculated by subtracting ΔCt of genes in fresh-isolated PBMCs from the ΔCt of genes in WNV- or mock-inoculated PBMCs at each time-point (for WNV-stimulated PBMCs, ΔΔCt_WNV_ = ΔCt_WNV_ − ΔCt_fresh_; and for mock-inoculated PBMCs, ΔΔCt_mock_ = ΔCt_mock_ − ΔCt_fresh_). A time-dependent relative expression patterns of **(B)** *TRAF3*, **(C)** *STAT1*, **(D)** *IRF7*, and **(E)***IRF9* mRNA in WNV-challenged rabbit PBMCs at different time points. Line graphs without common superscript differ significantly (*p* < 0.05). Upper case letter denotes difference of a gene expression among the time points in WNV-challenged cells; lower case letter denotes difference of a gene expression among the time points in mock-inoculated cells. *indicates the difference of a gene expression between WNV- and mock-challenged cells in the same time point (*p* < 0.05).

**Figure 5:**
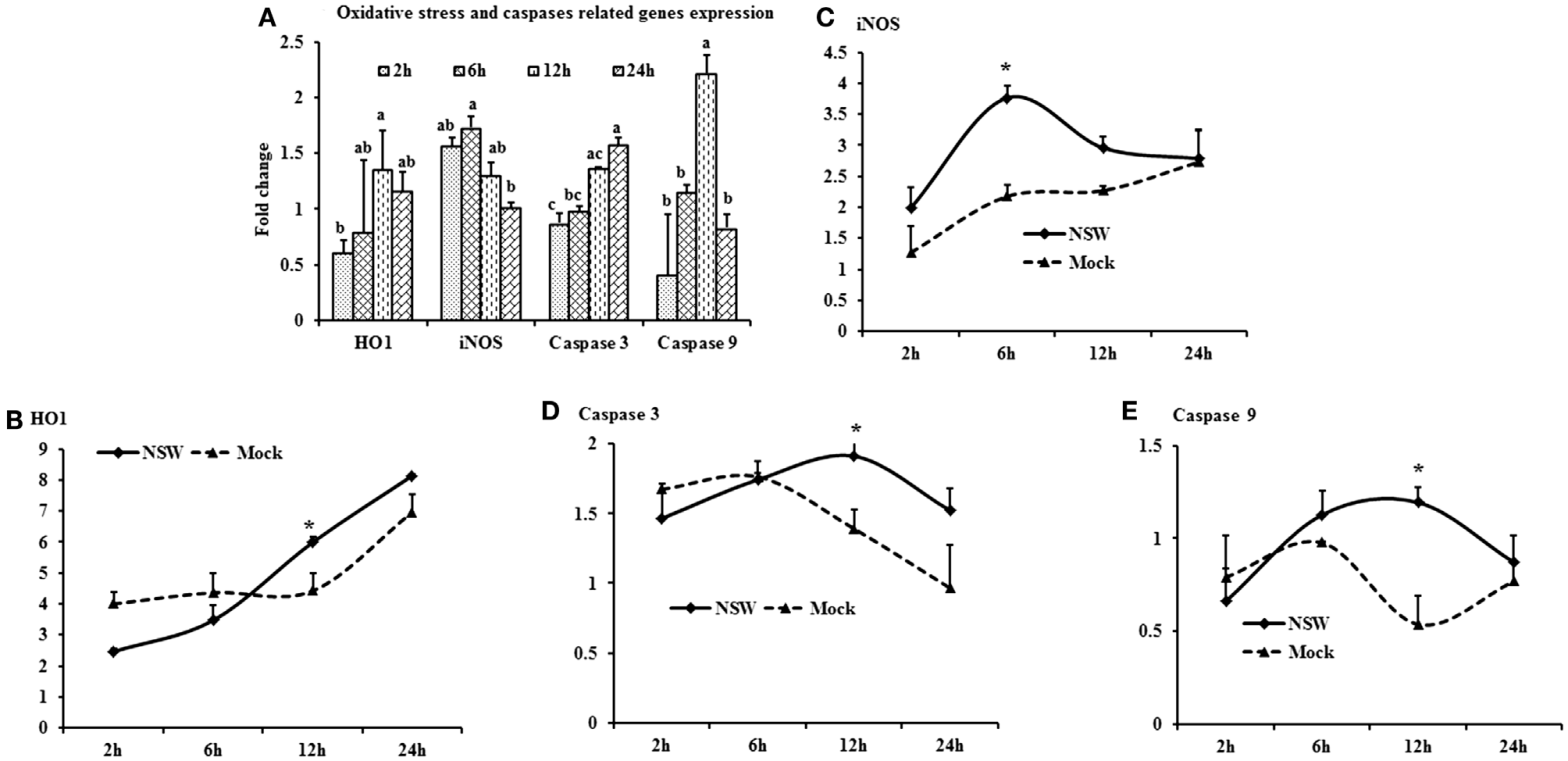
**Differential expression of oxidative stress and apoptosis-related genes in response to West Nile virus**. **(A)** Oxidative stress and apoptosis-related genes mRNA expression in fold change. The ΔΔCt (ΔΔCt = ΔCt_WNV_ − ΔCt_mock_) values were calculated by subtracting the ΔCt of genes in mock-inoculated PBMCs (*n* = 3). The bar graph showed the expression of genes in WNV-infected PBMCs over mock-inoculated PBMCs (fold change: the normalized expression value of a gene in WNV-stimulated cells/the normalized expression value of a gene in mock-inoculated cells). Bars without common superscripts (a,b; a,b,c) denote statistical difference of a gene expression among time points (*p* < 0.05). **(B–E)** Relative expression of oxidative stress and apoptosis-related genes mRNA, accounting for the effects of culture conditions on gene transcription in WNV- and mock-inoculated rabbit PBMCs (*n* = 3). To compare the normalized expression of oxidative stress- and apoptosis-related genes from PBMCs harvested at each time point to their respective expression levels before either WNV inoculation or mock inoculation, the ΔΔCt values were calculated by subtracting ΔCt of genes in fresh-isolated PBMCs from the ΔCt of genes in WNV- or mock-inoculated PBMCs at each time-point (for WNV-stimulated PBMCs, ΔΔCt_WNV_ = ΔCt_WNV_ − ΔCt_fresh_; and for mock-inoculated PBMCs, ΔΔCt_mock_ = ΔCt_mock_ − ΔCt_fresh_). A time-dependent relative expression patterns of **(B)** *HO1*, **(C)** *iNOS*, **(D)** *Caspase 3* and **(E)** *Caspase 9* mRNA in WNV-challenged rabbit PBMCs at different time points. Line graphs without common superscript differ significantly (*p* < 0.05). Upper case letter denotes difference of a gene expression among the time points in WNV-challenged cells; lower case letter denotes difference of a gene expression among the time points in mock-inoculated cells. *indicates the difference of a gene expression between WNV- and mock-challenged cells in the same time point (*p* < 0.05).

To quantify the mRNA expression for target and reference genes, qRT-PCR was performed using the Rotor Gene Corbett 6000 quantitative real-time PCR system (Qiagen). A one step qRT-PCR was performed using Rotor-Gene SYBR Green RT-PCR Kit (Qiagen). Each run contained each RNA sample and a no-template control. qRT-PCR was set up using 1 μL of RNA template, 5.25 μL of deionized RNase free H_2_O, 0.5 μM of upstream and downstream primers, 0.25 μL Rotor-Gene RT mix (RT mix), and 12.5 μL of 2× Rotor-Gene SYBR Green RT-PCR master mix (MM) (Qiagen) in a total reaction volume of 20 μL. The cycling conditions included reverse transcription step (10 min at 55°C), PCR initial activation step (5 min at 95°C), and a two-step cycling protocol with a denaturation step and a combined annealing/extension step. The two-step thermal cycling conditions were 5 s at 95°C followed by 10 s at 60°C (40 cycles). An amplification-based auto-threshold (Rotor-Gene Q Series Software, Qiagen) and adaptive baseline were selected as algorithms. Melting curve analysis was performed to detect the specificity of the PCR reaction. Each sample was run twice, and the average value was used as expression value. The qRT-PCR products of selected genes (e.g., GAPDH) on agarose gel. The gel documentation showed amplification only at the anticipated product length (i.e., 126 bp for GAPDH) (data not shown). Gene-specific expression was measured as relative to the geometric mean of the expression of two normalizer genes (*GAPDH* and *PPIA*) (Table 1). The delta Ct (ΔCt) (ΔCt = Ct_target_ − Ct_normaliser genes_) values were calculated as the difference between target gene and reference genes, and expression was calculated as 2^(−ΔΔCt)^ (36). To compare the magnitude of gene expression, the fold change was calculated. For this purpose, the delta delta Ct (ΔΔCt) values were calculated as follows: ΔΔCt = ΔCt_WNV_ − ΔCt_mock_. The bar graphs (Figures 1–5A; Figure 7) show the expression of genes in WNV-stimulated PBMCs over mock-inoculated PBMCs (fold change: the normalized expression value of a gene in WNV-stimulated cells/the normalized expression value of a gene in mock-inoculated cells). In addition, accounting for the effects of culture conditions on gene transcription in WNV- and mock-inoculated rabbit PBMCs, the normalized expression of genes from PBMCs harvested at each time point was compared to their respective expression levels before either WNV inoculation or mock inoculation. The ΔΔCt values were calculated by subtracting ΔCt of genes in fresh-isolated PBMCs from the ΔCt of genes in WNV- or mock-inoculated PBMCs at each time-point. The average expression values of the mRNA levels were considered for further analysis.

### Statistical Analysis

The technical replications were averaged. The impact of virus-challenge (treatment) and duration of incubation (time points) were evaluated using the SAS software package v. 9.2 (SAS Institute, Cary, NC, USA). For this purpose, the GLM (general linear model; Proc GLM) procedure and the implemented analysis of variance (ANOVA) statistic were used. Pairwise comparisons were performed between the time points and treatment groups using Tukey’s multiple comparisons in SAS, where *P* value was simultaneously adjusted. Besides, student’s *t*-test was applied when treatment groups were compared. The data were expressed as means ±SD and values of *p* < 0.05 were considered to indicate statistically significant differences.

## Results

### Expression Dynamics of Cytokines

The mRNA levels of cytokine *IFNA*, *IFNB*, *IFNG*, *IL6*, *IL12*, *CXCL10*, *IL22*, and *TNFA* showed a time-dependent expression pattern in rabbit PBMCs in response to WNV infection (Figures 1A and 2A). When gene expressions in WNV-infected PBMCs were expressed in fold change with regards to the expression of genes in control (mock-inoculated) PBMCs (Figures 1A and 2A), *IFNA* expression was up-regulated (8.4-folds) between 2- and 6-h pi and then declined (Figure 1A). The highest expression of *IFNB* gene was detected at the beginning of PBMCs–virus interaction and then declined gradually over time (Figure 1A). *IFNG* mRNA expression was increased over time and peaked (4.6 times) at 12-h pi (Figure 1A). While *IL6* and *IL22* mRNA expression increased over time, pentraxin 3 (*PTX3*) expression was significantly increased at the earlier hours of virus stimulation (Figure 2A).

When cytokine mRNA expressions in WNV-infected and mock-inoculated PBMCs were calculated with regards to the expression in freshly isolated PBMCs (fresh-PBMCs), *IFNA* mRNA expression was significantly higher at 6 h, then declined to the expression level in control at 12-h pi (Figure 1B). *IFNB* mRNA showed similar expression pattern in both infected and control cell lines (Figure 1C). With the exception at initial cell–pathogen interaction, *IFNG* (Figure 1D) and *TNFA* (Figure 2B) mRNA expression in virus-infected PBMCs was greater than in the mock-inoculated PBMCs. *IL12* expression in both the infected- and mock-inoculated PBMCs exhibited a similar pattern of expression, peaking at 6-h pi then declined (Figure 2C). *PTX3* gene expression was significantly up-regulated in the early hours of WNV infection, then declined to the expression level in mock-inoculated PBMCs (Figure 2D).

### Expression Patterns of TLRs and Associated Genes

The TLR-family genes showed similar patterns of expression characteristics with higher genes involvement at the beginning as well as at 24 h of post-virus stimulation, except *TLR2* and *TLR6* (Figure 3A). *TLR3*, *4*, and *6* mRNA expressions was higher in virus-stimulated PBMCs, compared to the mock-inoculated PBMCs (Figure 3A). When mRNA expressions in WNV-stimulated and mock-inoculated PBMCs were calculated with regards to the expression in fresh PBMCs, *TLR3* and *TLR4* expression was up-regulated at the initial hour pi in virus-stimulated PBL compared to mock-inoculated PBMCs, then declined (Figures 3B,C). *TLR10* mRNA expression was up-regulated in both WNV-stimulated and mock-inoculated PBMCs (Figure 3D).

When mRNA expressions in WNV-stimulated PBMCs were expressed in fold change with regards to the expression of mRNA in mock-inoculated PBMCs, *MyD88* expression was initially up-regulated then declined, whereas *IRF7* mRNA expression increased over time (Figure 4A). *STAT1* mRNA expression peaked at 24-h pi in WNV-stimulated PBMCs compared to mock-inoculated PBMCs (Figure 4A). *TRAF3* mRNA expression increased over time, peaking at 24-h pi (Figure 4B), and *STAT1* mRNA expression was significantly up-regulated at 24-h pi (Figure 4C) in virus-stimulated PBMCs. *IRF7* mRNA expression between the earlier and later hours of stimulation showed opposite pattern in response to WNV stimulation in rabbit PBMCs (Figure 4D). *IRF9* showed a similar pattern of expression both in WNV-stimulated and mock-inoculated PBMCs with a significant upregulation at 12-h pi (Figure 4E).

### Oxidative Stress and Apoptosis-Related Genes Expressions

Oxidative stress-related *HO1* gene expression became up-regulated over time, whereas iNOS mRNA expression decreased over time after a slight peak at 6-h pi in virus-stimulated PBMCs (Figure 5A). The expression of the apoptosis-associated gene *caspase 3* increased over time, whereas *caspase 9* mRNA expression was up-regulated at initial hours pi, then declined (Figure 5A).

When mRNA expressions in WNV-stimulated and mock-inoculated PBMCs were calculated with regards to the expression in fresh-PBMCs, *HO1* mRNA expression increased over time in both WNV-stimulated and mock-inoculated PBMCs (Figure 5B), whereas *iNOS* expression was higher in virus-stimulated PBMCs at 6-h pi (Figure 5C). Both *caspase 3* and *9* mRNA expressions were higher in virus-stimulated PBMCs at 12-h pi, then declined (Figures 5D,E).

### Kinetics of Virus-Specific Gene Expression in Rabbit PBMCs

**Figure 6:**
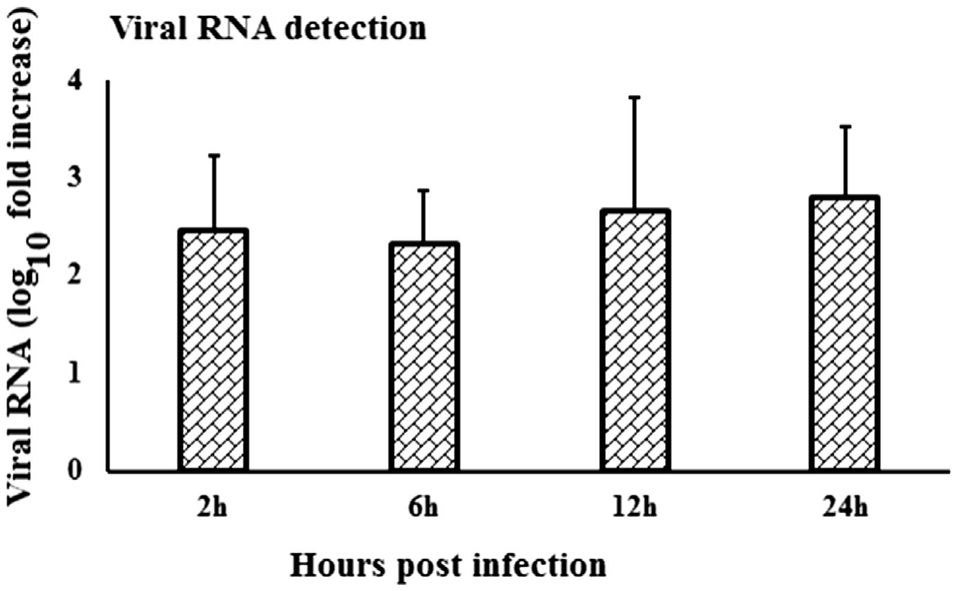
**Kinetics of viral RNA expression in *in vitro* WNV-infected PBMCs**. *WNV_NSW2011_* RNA expression in *in vitro* virus-infected PBMCs at different time points was been quantified using qRT-PCR. The ΔΔCt (ΔΔCt = ΔCt_WNV_ − ΔCt_mock_) values were calculated by subtracting the ΔCt of genes in mock-inoculated PBMCs (*n* = 3). Ct for the mock-inoculated PBMCs (undetected) was set to 40. The bar graph showed the expression of genes in WNV-infected PBMCs over mock-inoculated PBMCs (fold change: the normalized expression value of a gene in WNV-stimulated cells/the normalized expression value of a gene in mock-inoculated cells).

When viral RNA expression was compared among the virus-stimulated PBMCs samples, the WNV-specific transcript expression was increased at 24-h pi compared to the expression at 2- and 6-h pi (Figure 6). Notably, viral RNA could not be detected in *in vitro* mock-inoculated PBMCs or in fresh PBMCs samples or in PBMCs collected from *in vivo* virus infected or control rabbits.

### Validation of Transcripts Expressions in PBMCs from Virus-Infected Rabbits

**Figure 7:**
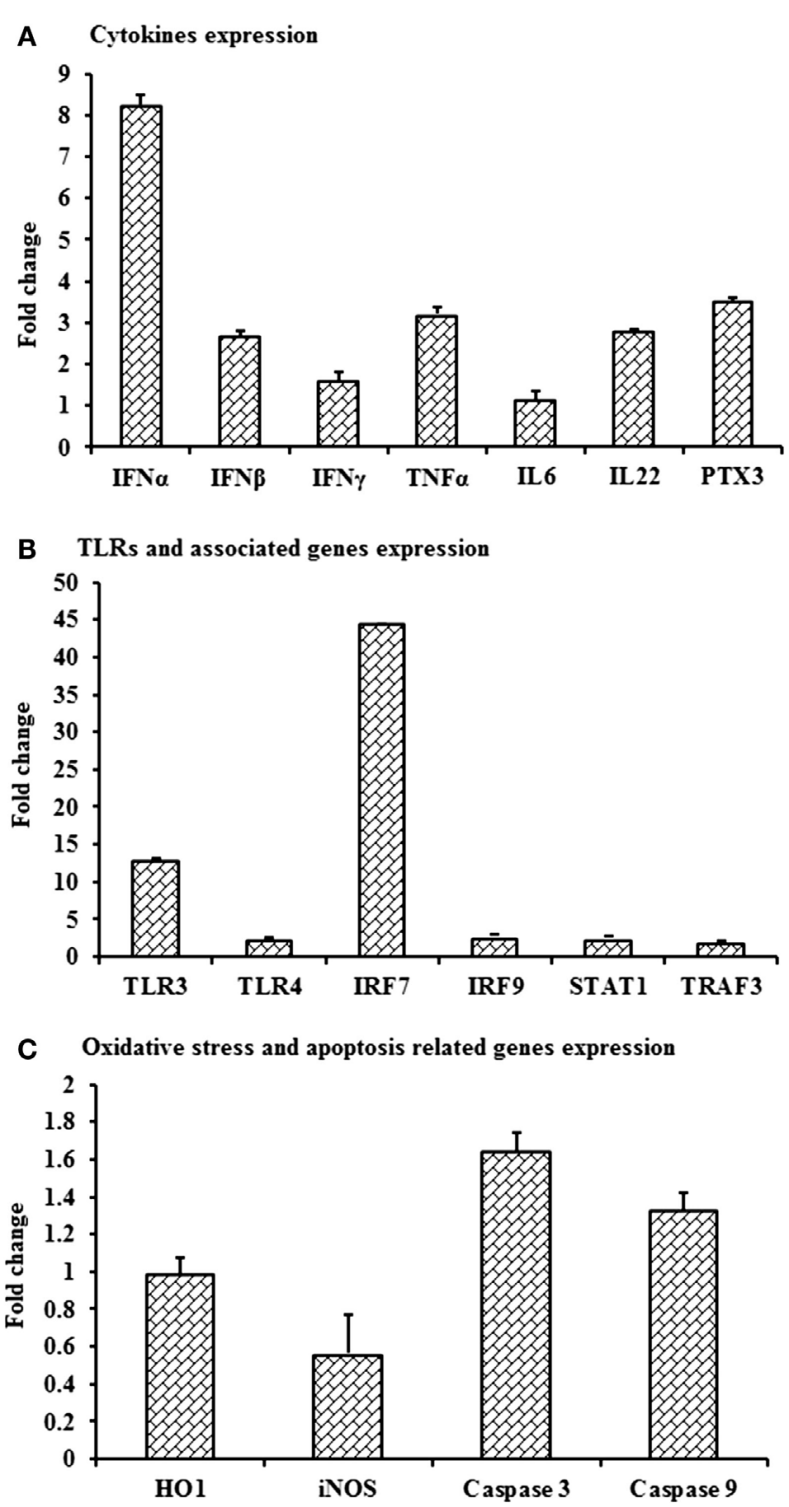
**Differential expression of mRNA level of selected immune molecules in *in vivo* WNV-infected rabbits**. Expression of **(A)***IFNA*, *IFNB*, *IFNG*, *TNFA*, *IL6*, *IL22* and *PTX3*, **(B)** *TLR3*, *TLR4*, *IRF7*, *IRF9*, *STAT1*, and *TRAF3*, and **(C)** *HO1*, *iNOS*, *Caspase 3*, and *Caspase 9* mRNA in WNV_NSW2011_-infected rabbit PBMCs at day 3 post-inoculation in fold change. The ΔΔCt (ΔΔCt = ΔCt_WNV_ − ΔCt_mock_) values were calculated by subtracting the ΔCt of genes in uninfected control rabbit PBMCs (*n* = 3). The bar graph showed the expression of genes in WNV-infected PBMCs over uninfected control rabbit PBMCs (fold change: the normalized expression value of a gene in *in vivo* WNV-infected rabbit cells/the normalized expression value of a gene in uninfected control rabbit cells). Data are presented as mean ± SD.

Despite the lack of detectable viral RNA in PBMCs collected on day 3 pi from WNV_NSW2011_-infected rabbits, the cells had upregulation of *IFNA*, *IFNB*, *TNFA*, *IL22* and *PTX3* (Figure 7A), *TLR3* and *IRF7* (Figure 7B), and *caspase 9* (Figure 7C) mRNA expression when compared to PBMCs from mock-infected control rabbits. *IFNA*, *IFNB*, *TNFA*, and *PTX3* mRNA expression was 8-, 2.8-, 3.2-, and 3.5-folds, respectively, higher in PBMCs from the virus-infected rabbits compared to the PBMCs from the control animals (Figure 7A). Remarkably, *TLR3* and *IRF7* transcripts were up-regulated by 13 and 44 times, respectively, in PBMCs from WNV_NSW2011_-infected rabbits compared to PBMCs from mock-infected rabbits (Figure 7B).

## Discussion

The economic burden of non-lethal WNV disease in horses and humans is substantial (37–39). Due to the cost and logistic limitations of using horses for pathogenesis studies, we have recently established an alternative small animal model in laboratory NZW rabbits to study the host–pathogen interactions (25). Tracking changes in the gene expression following viral infection is paramount to understand the host–pathogen interactions including the host–immune responses and pathogenesis. In this study, the expression patterns of selected genes involved in the innate immune response are documented in rabbit blood mononuclear cells following *in vitro* and *in vivo* challenge with an equine-virulent, Australian strain of WNV. The innate immune component includes Toll-like receptors, acute phase proteins, and cytokines expressed by different cell types including blood leukocytes. Among the ten members of the TLR family (TLR1-10), TLR3, 7, 8, and 9 are reported to recognize viral genomic components (40). TLR3 has been associated with the direct recognition of double-stranded viral RNA, while TLR7 and TLR8 target single-stranded viral RNA (40). The involvement of TLR3 (12) and TLR7 (18) have been extensively studied for WNV infection and recognition in mice (15), however, TLR7 and TLR8 are reported to be absent and pseudogenized, respectively, in rabbit (*O. cuniculus*) (41). Furthermore, TLR9 recognizes unmethylated viral-CpG DNA leaving TLR3 the only available TLR for the recognition of viral RNA in the rabbit. In case of WNV infection, peripheral inflammatory responses are initiated through the TLR3 (11, 15). In this study, upregulation of *TLR3* mRNA in *in vitro* virus challenged rabbit PBMCs was detected, suggestive of the involvement of this molecule in the WNV-induced innate immune response.

Although there was only a marginal and transient upregulation of the expression of *MyD88* mRNA under the present culture conditions, a study documented that MyD88 is involved in the restriction and spread of WNV in mice (16). *MyD88-*deficient mice showed elevated viral burden, and increased WNV replication was observed in *MyD88* deficient macrophages and subsets of neurons in cell culture [reviewed by Ref. (11)]. This implies that WNV, after being recognized by TLRs, initiates the downstream signaling cascades of the MyD88 and TRAF dependent pathways. Even in the face of some disparity in the utilization of adaptor proteins by different TLRs, their downstream signaling cascades converge on the transcription factor NFκB. TLR engagement initiates rapid signaling events that lead to activation of the transcription factors NFκB and IRF3, and production of cytokines including type I IFNs (11). The upregulation of *IFNA* was only transient in the *in vitro* WNV-stimulated PBMCs compared to mock-inoculated PBMCs. Induction of IFNα genes occurs mainly via the transcriptional activity of IRF7 (11). Recently, over expression of *TLR2*, *TLR3*, *TLR5*, *MyD88*, *STAT1*, *CXCL10*, *IL6*, *IL12*, and *TNFA* has been quantified in various tissues collected from *in vivo* WNV-infected mice (26). Higher mRNA expression of adaptor molecule MyD88, STAT1, TRAF3, IRF7, and 9 is suggestive of the involvement of these molecules in the rabbit PBMCs innate immune response to WNV. STAT1 is the key molecule controlling the course of IFN stimulation and kinetics of ISG expression. The type I IFN response depends on the phosphorylation patterns of STAT1 and is important for the WNV-induced immune response (42). Our results corroborate this, as *STAT1* mRNA increased over time in the *in vitro* WNV-infected rabbit PBMCs. WNV has been reported to block the phosphorylation of STAT1 as a means of immune evasion (11), leading to the blocking of IRF9 expression. However, IRF9 mRNA expression was up-regulated in *in vitro* WNV-stimulated rabbit PBMCs, suggesting that the reported immune evasion mechanisms either do not occur in rabbit PBMCs or at least not under the present culture conditions.

Productive replication of WNV has previously been demonstrated in *in vitro-*infected horse PBMCs by viral growth curve and qRT-PCR for WNV RNA (10). These authors reported that peak virus titer was reached at 6-day pi and high titers were maintained through 10- to 15-day pi (10). Rawle et al. (43) compared the growth kinetics of WNV_NSW2011_ and WNV_NY99_ using plaque assay and found that WNV_NSW2011_ did not replicate in human blood monocyte-derived dendritic cells as they extended the experiment from 24- to 72-h pi, whereas WNV_NY99_ successfully replicated in these cells during the entire duration of the experiment. Since other studies (10, 43) did not quantify viral RNA during the first 24-h pi, a comparison to our findings of viral RNA detection is precluded. These differences in growth kinetics in leukocytes between WNV_NSW2011_ and WNV_NY99_ may be ascribed to virus characteristics *sensu stricto* or may be explained by their respective ability to induce protective innate immune responses. In case of *in vivo* NZW rabbit infection, the draining lymph node was found to be the main site for peripheral replication of WNV_NSW2011_ following foodpad inoculation, with peak levels reached on day 3 pi (25). In addition, virus antigen was detected in pleomorphic leukocytes, macrophages, and/or dendritic cells in the paracortical zone of draining popliteal lymph nodes and in the leukocytes in multiple sites of the deep dermis of the injected footpad (25). It is important to note that the present study was limited to 24 h post-*in vitro* stimulation of rabbit PBMCs, as the study aimed to decipher the patterns of early responses of selected genes involved in innate immune response to WNV. Further studies will be required to characterize the virus growth kinetics in rabbit PBMCs over extended time periods but that was beyond the scope of the present study.

Higher expression of IRF7 mRNA *in vitro* stimulation of PBMCs and in PBMCs from rabbits 3 days post-*in vivo* infection is suggestive of its involvement in WNV-induced innate immune responses in the rabbit. TLR3 activation leads to the induction of IRF7 which triggers IRF3 and NFκB to produce IFNs (11). A deficiency of *IRF7* completely abrogated the *IFN*α response while no effect on *IFNB* gene induction was observed in IRF7−/− macrophages in mouse (13). *IFNB* mRNA expression was unaffected in *in vitro* virus-induced PBMCs, which may be explained by the previous findings that *IRF3* and *IRF7* only partially regulate the *IFNB* gene and ISG expression in macrophages (13). The upregulation of *IRF7* and IFNA mRNA expression was significantly higher in *in vitro* WNV-stimulated rabbit PBMCs compared to mock-inoculated PBMCs, suggesting the involvement of these downstream molecules in WNV infection.

Unexpectedly, mRNA expression of *TLR4* and *TLR6*, which recognizes bacterial pathogens, was found to be significantly up-regulated in this study. Antiviral activity of TLR3 and TLR4 has been detected in human microglial cells (44). Single-stranded RNA viruses such as respiratory syncytial virus (RSV) are documented to activate the innate immune response through TLR2 and TLR6 in murine macrophages (45). However, an irregular pattern of *TLR2* expression was found in WNV-stimulated rabbit PBMCs. Nevertheless, this is in agreement with previous studies that demonstrated varying TLR expression in different cell types (46). TLR2 is reported to recognize Epstein–Barr virus (EBV) in human monocyte (47). It is important to note that RSV and EBV are negative sense ssRNA and DNA virus, respectively, whereas WNV is a positive sense ssRNA virus. Recently, upregulation of TLR2 and TLR3 within 1-day pi has been reported in WNV-infected mouse tissues (26), which coincided with our findings in PBMCs.

Recognition of WNV through TLR signaling pathways via MyD88 and TRIF adaptor molecules induced the IRF family and NF-κB genes. NF-κB binds to transcription sites and induces an array of genes that are responsible for production of acute phase proteins, iNOS, coagulation factors, and pro-inflammatory cytokines. Some of the major immune pathways identified to be up-regulated by microarray in the equine brain following experimental WNV infection included the *IL15*, *IL22*, *IL9*, and *IFN* signaling pathways (19), while in mice *IFNB*, *TNFA*, and *IL6* may be key factors (17). In this study, *TNFA* mRNA was up-regulated in *in vitro* WNV-stimulated rabbit PBMCs, suggesting that *TNFA* may be an important component of the WNV-induced innate immune response. Although rabbit PBMCs have not been studied before, the role of macrophages in WNV infection has been reviewed earlier (11). Activation of macrophages in response to WNV infection also promotes the release of type I IFN, TNFA, IL1B, IL8, and other cytokines, thus reducing viral replication in cell culture [reviewed by Ref. (11)]. TNFα is strongly induced by TLR activation and consequently, cellular activation by TNFα could potentially induce TLR gene expression and provide a means for enhancing cellular responsiveness to microbial ligands recognized by those TLRs (40). TNFα mRNA expression was found to be up-regulated over time and peaked at 24-h pi, which is similar to findings by Kwon et al. (48), who found that *TNFA* was significantly up-regulated in horse monocytes 12 and 20 h after challenge with synthetic poly I:C.

*IFNG* mRNA expression was up-regulated from 6 to 24 h after *in vitro* WNV stimulation of rabbit PBMCs. A dominant protective antiviral role of IFNG against WNV has been documented to occur in peripheral lymphoid tissues (49). A notable difference in the levels of type I and II interferon was reported in the brain in WNV_NSW2011_-infected rabbits, but their expressions were invariable in draining lymph nodes (25). A lack of IFNG production or signaling was reported to increase vulnerability to lethal WNV infection in mice, with a rise in mortality, a decrease in survival time, higher viremia and greater viral replication in lymphoid tissues (49). γδT cells require IFNG to limit the dissemination of WNV and treatment of primary dendritic cells with IFNG-reduced WNV replication (49). However, it remains to be determined which cell subset in the rabbit PBMCs were responsible for the IFNG mRNA expression. *IL22* mRNA was up-regulated over time in rabbit PBMCs in response to *in vitro* WNV stimulation. IL22 is expressed by a wide range of immune cells, including T and NK cells, and engagement of the IL22 receptor leads to STAT3 and STAT1 signaling (50). Notably, relatively high expression of *IL22* mRNA by rabbit PBMCs following *in vivo* infection of the animals may help explain that no viral RNA was detected in their PBMCs at 3-day pi. The expression of *IL22* was consistent with a previous study detecting upregulation of *IL22* mRNA in WNV infected horse lymphoid tissues (19).

*PTX3* was one of the most (4.2-folds) up-regulated genes in rabbit PBMCs infected with WNV *in vitro*. Microarray expression analysis showed that *PTX3* was the gene displaying the most pronounced expression in thalamus and cerebrum of horses experimentally infected with WNV (19). PTX3 is a soluble, acute phase protein (soluble pattern recognition receptor; PRMs) and recognizes PAMPs (51). PTX3 is produced by a variety of cells and tissues, most notably dendritic cells and macrophages, in response to TLR engagement and inflammatory cytokines. This molecule has many functions, including an integral role in the pathway of PRRs in recognition of viruses and bacteria (52). The *PTX3* gene is induced by *IL1B* and *TNFA*, and functions in phagocytosis and opsonization of antigens, as well as in the inflammatory response (52). Human and murine PTX3 bound to influenza virus and mediated a range of antiviral activities, including inhibition of hemagglutination, neutralization of virus infectivity, and inhibition of viral neuraminidase (53). The exact role of *PTX3* in WNV-infection remains to be elucidated, but our results suggest a potential antiviral role in some species, notably rabbit (Figure 2).

Apoptosis is a highly conserved mode of programed cell death, mediated by the activation of caspases. WNV is reported to induce apoptosis in human brain derived glia cells in culture by the activation of caspase 3, 8, and 9 (54). WNV proteins such as envelope (E) and non-structural protein 3 (NS3) have been shown to induce caspase-dependent apoptosis when transfected into cells (55). Apoptosis is induced through the mitochondrial pathway resulting in caspase 9 and caspase 3 activation in mouse brain cells *in vitro* (55). WNV NS3 induced host cell apoptotic pathways involving caspase 8 and 3 in different cell types (56), but so far there has been no study of caspase expressions either in rabbit or equine PBMCs following WNV infection. Higher expression of *caspase 3* and *9* mRNA both in *in vitro-*infected rabbit PBMCs and in PBMCs from the *in vivo* rabbit model are in accordance with other studies establishing that caspase 3, 8, and 9-dependent apoptosis is involved in WNV infection. It remains to be shown that the rabbit cells proceed to undergo apoptosis following WNV exposure *in vitro*.

*HO1* mRNA expression was increased at 12- to 24-h post-WNV infection of PBMCs *in vitro*, whereas iNOS mRNA expression was up-regulated at 6-h pi. In contrast, neither *iNOS* nor *HO1* appeared to be affected in the PBMCs from WNV-infected rabbits. The discrepancy might be due to the time difference between the *in vitro* and the *in vivo* study. Monocytes infiltrating into the brain of mice in WNV-induced encephalitis produced nitric oxide (NO) (57). Macrophages have been reported to control JEV infection directly through the production of NO and other reactive oxygen intermediates (58). Activation of HO1 by a natural substrate, hemin, effectively enhanced the ability of human macrophages to resist infections by several pathogens, including dengue virus, WNV and poxvirus (59). Similarly high expression of *TNFA* and *HO1* might suggest that oxidative stress protects rabbit PBMCs from WNV infection both *in vitro* and *in vivo*.

## Conclusion

Expression patterns of selected genes involved in the innate immune response to WNV have been documented in this study using rabbit PBMCs as an *in vitro* model. Expression of selected genes was validated in the WNV-infected rabbit *in vivo*. A rabbit model has several advantages over the mouse model, the more commonly used model for WNV, by mimicking the course of infection in the horse better, including viremia, virus distribution, and morbidity. Therefore, the presented data on the genes pivotal in WNV infection in a novel rabbit cell model will help to focus on candidate markers for further study. Specifically, a pan-genomic approach using Next-Generation Sequencing would have yielded much deeper insights into the differential expression in response to WNV.

## Author Contributions

MU conceived and designed the experiments, performed the experiments, analyzed the data, drafted, and edited the manuscript. WS conceived and designed the experiments, performed the experiments, analyze the data, and edited the manuscript. NP and RH conceived and designed the experiments and edited the manuscript. HB-O conceived and designed the experiments, analyzed the data, drafted, and edited the manuscript.

## Conflict of Interest Statement

All authors read and approved the final manuscript. The authors declare that they have no competing interests.

## Abbreviations

ACTB, actin, beta; CXCL10, C–X–C motif chemokine 10 [also known as Interferon gamma-induced protein 10 (IP-10)]; GAPDH, glyceraldehyde 3-phosphate dehydrogenase; HO1, heme oxygenase 1; IFNα, interferon alpha; IFNβ, interferon beta; IFNγ, interferon gamma; IL12, interleukin IL22; IL6, interleukin 6; iNOS, inducible nitric oxide synthase; IRF, interferon regulatory factor; Kunjin, australian strain of west nile virus; MyD88, myeloid differentiation 88; PPIA, peptidylprolyl isomerase A (also known as cyclophilin A); PTX3, pentraxin 3; STAT1, signal transducer and activator of transcription 1; TLR, toll-like receptor; TNFα, tumor necrosis factor alpha; TRAF3, TNF receptor-associated factor 3.
