# Supplementary material for: West Nile Virus Challenge Alters the Transcription Profiles of Innate Immune Genes in Rabbit Peripheral Blood Mononuclear Cells
Source: Front Vet Sci. 2015 Dec 14;2:76. doi: 10.3389/fvets.2015.00076 (PMC4677099; doi:10.3389/fvets.2015.00076)
Supplement: Supplementary file 1 [file Table_1.DOCX]

Supplementary file 1. **Effect of duration of incubation (time in h) and WNV-stimulation on the relative expression of genes analysed by Proc GLM (SAS).**

| Gene | WNV-infection | Duration of incubation (h) | R^2^ | Model |
| --- | --- | --- | --- | --- |
| *IFNα* | ns | * | 0.51 | * |
| *IFNβ* | ns | *** | 0.65 | *** |
| *IFNγ* | * | ns | 0.34 | * |
| *TNFα* | ns | ns | 0.23 | ns |
| *IL6* | ns | ** | 0.56 | * |
| *IL12* | ns | ns | 0.27 | ns |
| *IL22* | ns | ns | 0.18 | ns |
| *CXCL10* | ns | ** | 0.59 | ** |
| *PTX3* | * | ns | 0.29 | * |
| *HO1* | ns | * | 0.48 | * |
| *iNOS* | ns | ns | 0.21 | ns |
| *Caspase 3* | ns | ns | 0.24 | ns |
| *Caspase 9* | ns | ns | 0.15 | ns |
| *TLR1* | * | * | 0.37 | * |
| *TLR2* | ns | ** | 0.39 | * |
| *TLR3* | ns | ** | 0.69 | ** |
| *TLR4* | ns | ns | 0.11 | ns |
| *TLR6* | ns | * | 0.43 | * |
| *TLR10* | ns | *** | 0.61 | *** |
| *MyD88* | ns | ns | 0.21 | ns |
| *STAT1* | * | ns | 0.34 | * |
| *TRAF3* | ns | *** | 0.42 | *** |
| *IRF1* | ns | ns | 0.18 | ns |
| *IRF7* | ns | ns | 0.20 | ns |
| *IRF9* | ns | * | 0.39 | * |

ns, not significant. *, *p* < 0.05; **, *p* < 0.01; ***, *p* < 0.001.
